# Left Atrial Appendage Occlusion Versus Oral Anticoagulants in Older Atrial Fibrillation Patients

**DOI:** 10.1093/geroni/igaf122.3387

**Published:** 2025-12-31

**Authors:** Xiaojuan Liu, Sebastian Schneeweiss, Daniel Singer, Jerry Avorn, E Kevin Heist, Joshua Lin

**Affiliations:** Brigham and Women’s Hospital, Boston, Massachusetts, United States; Brigham and Women’s Hospital, Boston, Massachusetts, United States; Massachusetts General Hospital, Boston, Massachusetts, United States; Brigham and Women’s Hospital, Boston, Massachusetts, United States; Massachusetts General Hospital, Boston, Massachusetts, United States; Brigham and Women’s Hospital, Boston, Massachusetts, United States

## Abstract

Real-world evidence comparing left atrial appendage occlusion (LAAO) devices to oral anticoagulants (OAC) in nonvalvular atrial fibrillation (NVAF) remain limited. We emulated a target trial and evaluated the effectiveness and safety of LAAO device versus OAC in 35326 NVAF patients (aged ≥65 years, CHA2DS2-VASc score ≥2 [males] or ≥ 3 [females]) using Medicare 2016-2020 and Optum 2016-2024 data. LAAO recipients on OAC (warfarin, apixaban, rivaroxaban, or dabigatran) at implantation (index date) were 1:1 matched to patients who received these medications alone via propensity score matching based on 75 pre-treatment covariates. Outcomes included hospitalization for major bleeding events, ischemic stroke, and all-cause mortality. Follow-up was censored at 2 years or upon treatment deviation (OAC discontinuation or LAAO implantation in OAC users and OAC continuation beyond 90 days in LAAO users). Database-specific rate ratios (RR) were estimated via Poisson regression with inverse probability of censoring weighting and pooled using fixed-effect meta-analysis. Compared to OAC users, LAAO users had a higher bleeding rate over 2 years (RR 1.29; 95% CI 1.11-2.49); however, beyond 6 months post-implantation, LAAO users had a 36% reduction in bleeding rate (0.64; 0.51-0.80). LAAO was also associated with a higher rate of ischemic stroke over 2 years (1.43; 1.10-1.85), though the effect size diminished over time and was no longer statistically significant beyond 6 months (1.16; 0.84-1.62). We found no evidence of association with mortality. These findings suggest that LAAO may reduce long-term bleeding risk but may carry an early increased risk of bleeding and ischemic stroke compared to OAC.

